# Reconstruction of the Evolutionary Dynamics of A(H3N2) Influenza Viruses Circulating in Italy from 2004 to 2012

**DOI:** 10.1371/journal.pone.0137099

**Published:** 2015-09-02

**Authors:** Erika Ebranati, Elena Pariani, Antonio Piralla, Monica Gozalo-Margüello, Carla Veo, Laura Bubba, Antonella Amendola, Massimo Ciccozzi, Massimo Galli, Alessandro Remo Zanetti, Fausto Baldanti, Gianguglielmo Zehender

**Affiliations:** 1 Dipartimento di Scienze Biomediche e Cliniche “Luigi Sacco”, Sezione di Malattie Infettive, Università degli Studi di Milano, Milan, Italy; 2 Dipartimento di Scienze Biomediche per la Salute, Università degli Studi di Milano, Milan, Italy; 3 SS Virologia Molecolare, SC Virologia e Microbiologia, Fondazione IRCCS Policlinico San Matteo, Pavia, Italy; 4 Marqués de Valdecilla University Hospital, Santander, Spain; 5 Istituto Superiore di Sanità, Dipartimento Malattie Infettive, Parassitarie ed Immunomediate, Rome, Italy; University of Oxford, VIET NAM

## Abstract

**Background:**

Influenza A viruses are characterised by their rapid evolution, and the appearance of point mutations in the viral hemagglutinin (HA) domain causes seasonal epidemics. The A(H3N2) virus has higher mutation rate than the A(H1N1) virus. The aim of this study was to reconstruct the evolutionary dynamics of the A(H3N2) viruses circulating in Italy between 2004 and 2012 in the light of the forces driving viral evolution.

**Methods:**

Phylodinamic analyses were made using a Bayesian method, and codon-specific positive selection acting on the HA coding sequence was evaluated.

**Results:**

Global and local phylogenetic analyses showed that the Italian strains collected between 2004 and 2012 grouped into five significant Italian clades that included viral sequences circulating in different epidemic seasons. The time of the most recent common ancestor (tMRCA) of the tree root was between May and December 2003. The tMRCA estimates of the major clades suggest that the origin of a new viral strain precedes the effective circulation of the strain in the Italian population by 6–31 months, thus supporting a central role of global migration in seeding the epidemics in Italy. The study of selection pressure showed that four codons were under positive selection, three of which were located in antigenic sites. Analysis of population dynamics showed the alternation of periods of exponential growth followed by a decrease in the effective number of infections corresponding to epidemic and inter-epidemic seasons.

**Conclusions:**

Our analyses suggest that a complex interaction between the immune status of the population, migrations, and a few selective sweeps drive the influenza A(H3N2) virus evolution. Our findings suggest the possibility of the year-round survival of local strains even in temperate zones, a hypothesis that warrants further investigation.

## Introduction

Influenza A viruses are characterised by their rapid evolution, which allows them to generate new strains against which humans are not immune on such a regularly that they cause seasonal epidemics and occasionally global pandemics. Human influenza viruses are one of the major cause of morbidity and mortality worldwide, on average accounting for infections in 5–15% of the global population and about 500,000 deaths a year [1,2].

The gene fragment coding for hemagglutinin (HA) is particularly important because surface glycoprotein HA is the main target of the immune system, and mutations in the globular head region of the protein (residues 50–230 of HA1 according to H3 HA numbering) give rise to antigenic novelty, species adaptation, and viral transmission [3] Influenza pandemics are characterised by a change in the viral HA subtype due to the re-assortment or introduction of a new virus (antigenic shift), whereas influenza epidemics are characterized by the acquisition of point mutations in the viral HA1 domain encoding the major antigenic sites of HA protein that lead to serial antigenic changes (antigenic drift) [4].

Of the eighteen known subtypes of HA and eleven subtypes of neuraminidase (NA) in influenza A viruses [1,4], H3N2 and H1N1 are currently the major circulating subtypes and have been circulating together since 1977 [1]; however, A(H3N2) has a higher mutation rate than A(H1N1) [5,6], and there has been a high rate of antigenic drift in the human H3 subtype since its emergence in 1968.

The aim of this study was to reconstruct the evolutionary dynamics of the A(H3N2) influenza viruses circulating in Northern Italy between 2004 and 2012 in the light of the forces driving viral evolution.

## Materials and Methods

### Ethics statement

The respiratory samples were collected by sentinel practitioners, and anonymously analysed at the reference laboratories of the Italian Influenza Surveillance Network (Influnet: http://www.iss.it/iflu/) and in the framework of severe influenza A surveillance program in Lombardy region (DGR IX/1046, 22 Dec. 2010 and DGR 5988, 30 Jun 2011). This retrospective study was performed according to the guidelines of the Institutional Review Board on the use of biological specimens for scientific purposes in keeping with Italian law (art.13 D.Lgs 196/2003) and was approved by the Ethics Commitee of Fondazione IRCCS Policlinico San Matteo in Pavia, Italy. The work described here is a retrospective study performed on left over samples that were obtained as part of routine tests performed. No extra samples were obtained for this research. The retrospective analysis was anonymous. Therefore, informed consent (either written or verbal) was not required.

### Sequence dataset

A sequence dataset was constructed that included 202 HA gene sequences obtained from as many A(H3N2)-positive respiratory specimens (nasal or oropharyngeal swabs) collected within the framework of Influnet from outpatients with symptoms of influenza-like illness (ILI) and hospitalised patients suffering from severe respiratory syndrome in Northern Italy. The A(H3N2) HA sequences were collected between January 2004 and April 2012 (i.e. during eight consecutive influenza seasons).

A preliminary global phylogenetic analysis was also made of a larger dataset constructed by aligning the sequences of these 202 patient isolates with 307 other A(H3N2) HA sequences from isolates collected throughout the world and obtained from the National Center for Biotechnology Information (NCBI: http://www.ncbi.nlm.nih.gov) GenBank or the Global Initiative on Sharing All Influenza Data (GISAID) EpiFlu database (platform.gisaid.org/epi3/). Their identification numbers are listed in S1 Table.

### HA amplification and sequencing

The A(H3N2) HA gene was amplified directly from the clinical specimens. Total RNA was extracted from the respiratory samples using the Nuclisens easyMAG automated extraction kit (BioMerieux, Lyon, France), and the sequences were obtained by means of an RT-PCR assay specific for a 882 bp fragment (nt. 174–1,056) in the HA1 domain [7]. The PCR products were purified using a Microcon-100 microconcentrator in accordance with the manufacturer's instructions (Millipore, Bedford, MA, USA), and then sequenced using a BigDye Terminator Cycle-Sequencing kit (Applied Biosystems, Foster City, USA) and ABI Prism 3100 DNA sequencer (Applied Biosystems, Foster City, USA).

The sequences were deposited in GenBank under accession numbers: EU400224-EU400228, EU400232, EU400233, EU400238, EU400241-EU400245, EU400248-EU400250, EU400252, EU400253, EU400255, EU400256, EU400261, EU400264-EU400267; JX051171-JX051214; JX239567-JX239619; JX262014-JX262068; JX401311-JX401338.

### Likelihood mapping analysis

The phylogenetic signal of each sequence dataset was investigated by means of the likelihood-mapping analysis of 10,000 random quartets generated using TreePuzzle. All of the three possible unrooted trees for a set of four sequences (quartets) randomly selected from the dataset were reconstructed using the maximum likelihood approach and the selected substitution model. The posterior probabilities of each tree were then plotted on a triangular surface so that the dots representing the fully resolved trees fell at the corners and those representing the unresolved quartets in the centre of the triangle (star-tree) [8]. Using this strategy, which has been described in detail elsewhere [9], the data are considered unreliable for phylogenetic inference when more than 30% of the dots are in the centre of the triangle.

### Path-O-Gen analysis

The clock-like signal of the dataset was investigated using Path-O-Gen software (freely available at http://tree.bio.ed.ac.uk/software/pathogen/), which analyses the correlations between time and the root-to-tip distances of a tree constructed without assuming a molecular clock.

### Phylogenetic analysis

The sequences were aligned using CLUSTALW (integrated within the Bio-Edit sequence editor by Tom Hall, 2001; http://www.mbio.ncsu.edu/BioEdit/bioedit.html). The best-fitting nucleotide substitution model was estimated using JModeltest [10], and selected a GTR model [11] with gamma-distributed rates among sites.

The phylogenetic tree, model parameters, evolutionary rates and population growth were co-estimated using a Bayesian Markov Chain Monte Carlo (MCMC) method implemented in the BEAST package v.1.74 [12].

A strict clock and an uncorrelated log-normal relaxed clock model were both implemented under a GTR + G substitution model. A Bayes factor (BF, using marginal likelihoods) implemented in Beast selected the best-fitting models [13]. In accordance with [14], only values of 2lnBF ≥6 were considered significant. A less restrictive Bayesian skyline plot (BSP, a non-parametric piecewise-constant model) was used as coalescent prior. Two independent MCMC chains were run for 30 million generations (with sampling every 3,000^th^ generation), and were combined using the LogCombiner 1.74 included in the BEAST package. Convergence was assessed on the basis of the effective sampling size (ESS) after a 10% burn-in using Tracer software version 1.5 (http://tree.bio.ed.ac.uk/software/tracer/). Only ESS’s of ≥200 were accepted.

Uncertainty in the estimates was indicated by 95% highest posterior density (95% HPD) intervals.

The obtained trees were summarised in a maximum clade credibility tree using the Tree Annotator program included in the BEAST package, and the tree with the maximum product of posterior probabilities (maximum clade credibility: MCC) after a 10% burn-in was displayed using Figtree version 1.3.1) (http://tree.bio.ed.ac.uk/software/figtree/).

### Selection pressure

Tests for positive selection were conducted on the Datamonkey server [15] using the single-likelihood ancestor (SLAC), fixed-effects likelihood (FEL), internal branch fixed-effects likelihood (IFEL), mixed effects model of evolution (MEME), and fast unconstrained Bayesian approximation (FUBAR) methods, and the *dN/dS* ratios were calculated using the SLAC and FEL codon-based maximum likelihood approaches. SLAC counts the number of non-synonymous changes per non-synonymous site (*dN*) and tests whether it is significantly different from the number of synonymous changes per synonymous site (*dS*). FEL estimates the ratios of non-synonymous to synonymous changes for each site in an alignment [16]. The IFEL method is similar to FEL, but tests site-by-site selection for only along internal branches of the phylogeny. In order to avoid an excessive false-positive rate, sites with SLAC, FEL, IFEL and MEME *p*-values of <0.1 and a FUBAR posterior probability of >0.90 were accepted as candidates for selection. The property informed model of evolution (PRIME) was designed to take into account the biochemical properties of the amino acids: it works using the same conceptual frameworks as FEL and MEME but, unlike FEL and MEME, allows the non-synonymous substitution rate β to depend on which residues are being exchanged as well as on the site in question.

The selected positive sites were superimposed on the HA structure using PyMOL Molecular Graphics system, version 1.3 (Schrödinger, LLC), and strain A/Aichi/2/68(H3N2) (PDB code 3VUN).

## Results

### Likelihood mapping and root-to-tip regression analysis

The likelihood mapping of 10,000 random quartets showed that more than 87.5% were distributed at the corners of the likelihood map and only 9.8% in the central area, thus indicating that the dataset contained sufficient phylogenetic information in S1 Fig.

The analysis of the Bayesian tree obtained using MrBayes showed a very high level of correlation (R^2^ = 0.94) between time and root-to-tip genetic distance, thus suggesting a high level of clock-like signals and showing that sequences from 2003–2004 season were the closest to the root (S2 Fig).

### Global phylogenetic analysis

A preliminary analysis of the global dataset of 509 A(H3N2) isolates (202 from Italy and 307 from the rest of the world) showed that the Italian strains were included in a number of previously described and widespread A(H3N2) HA genetic clades (S3 Fig). Two genetic clades (corresponding to A/Wyoming/03/2003 and A/Wellington/01/2004) were identified in the 2003–2004 season, and were followed by A/California/07/2004 (2004–2005 and 2005–2006), A/Wisconsin/67/2005 (2005–2006 and 2006–2007), A/Brisbane/10/2007 (2006–2007 and 2008–2009), and A/Victoria/208/2009, which was present in all the isolates obtained during the 2010–2011 season, but mainly in those obtained during the 2011–2012 season.

Ten purely Italian subclades (present in between 3 and 15 isolates) were interspersed throughout the tree. Most of them became extinct in the same season in which they originated, only exception being a single highly significant subclade (pp = 0.8) identified in five isolates sampled in different seasons between 2007 and 2009.

In order to reconstruct the population dynamics of the A(H3N2) epidemics in Italy, we separately analysed the Italian isolates with a known month of isolation, and estimated the evolutionary rate.

### Evolutionary rate estimates

A strict and a relaxed (log-normal) molecular clock model were implemented under two less stringent Bayesian coalescent models: a classical Bayesian skyline plot (BSP) and a GMRF skyride. As a BF test showed that the relaxed clock was significantly better than the strict clock (2lnBF = 93.6) and the BSP did not fit the data any better than the GMRF skyride (BSP: 2lnBF = 9, not significant), the skyride coalescent and relaxed clock models were selected for the reconstruction of time-scale phylogeny and population dynamics. Under these conditions, the estimated mean evolutionary rate of the HA sequences analysed using both the restricted Italian and the larger global alignments was 3.8×10^−4^ subs/site/month (95% HPD: 2.7–4.7×10^−4^).

### Bayesian dated tree analysis

**Fig 1:**
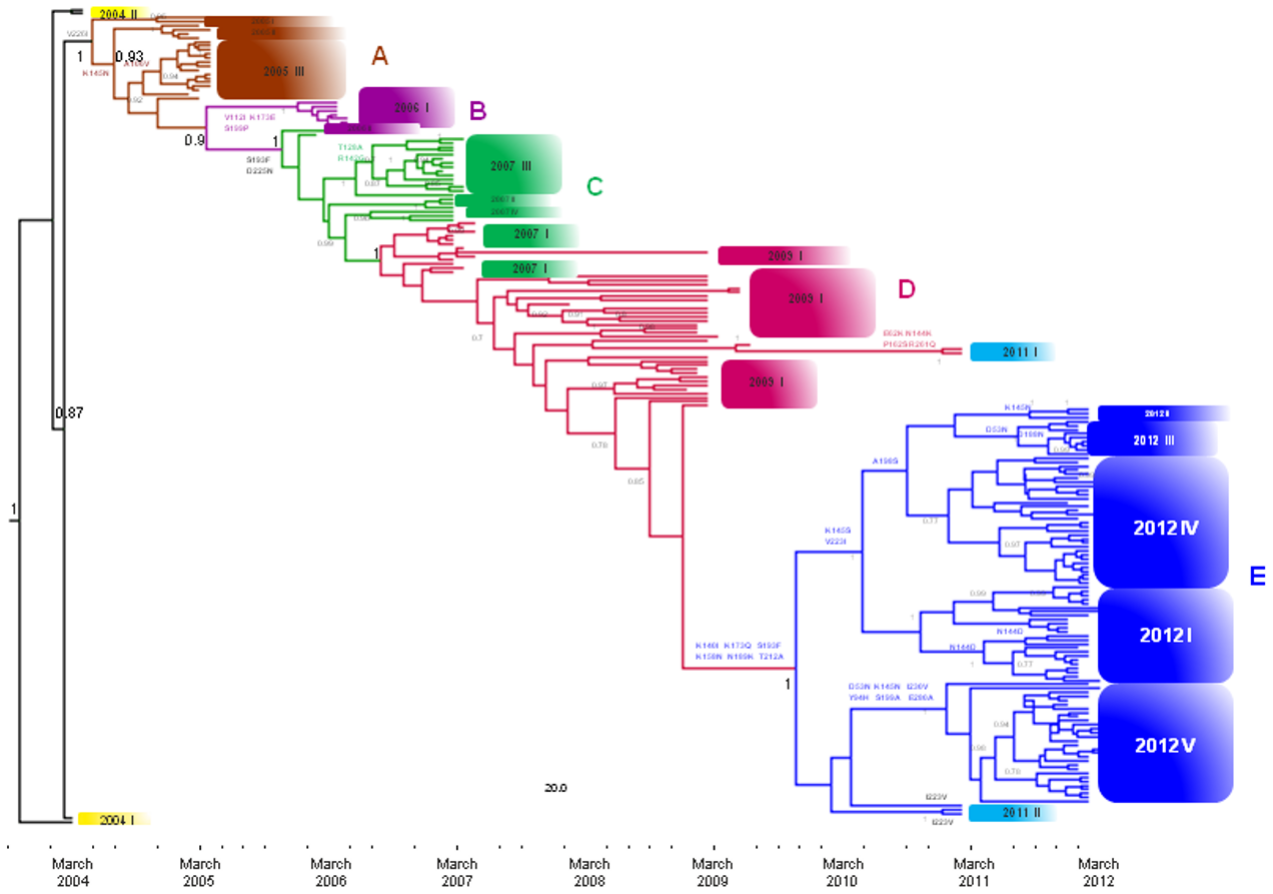
Bayesian maximum clade credibility (MCC) tree of the 202 Italian influenza A(H3N2) virus HA gene sequences. Different clusters and the Italian clades are indicated by different colours. The numbers on the internal nodes represent posterior probabilities. Amino acid substitutions characterising a particular branch are indicated. The scale at the bottom of the tree represents the calendar months between the tMRCA of the tree root and the most recent samples (March 2012).

A Bayesian time-scaled tree reconstructed using the Italian isolates (Fig 1) showed that the Italian strains segregated into at least five significant clades (Table 1) that included viral sequences circulating in different epidemic seasons.

**Table 1:** Amino acid substitution characterizing the Italian clusters.

YEARS	CLUSTERS	ITALIAN CLADES	MUTATIONS
**2005**			
	I	A	H183L; V226I
	II	A	K145N; V226I
	III	A	A106V^a^; K145N; V226I
**2006**			
	I	B	V112I; K173E; S199P;
	II	C	S193F^b^; D225N
**2007**			
	III	C	T128A^a^; R142G; L157S; K173E^a^; S193F^b^; D225N
	II	C	T128A; S193F^b^; D225N
	IV	C	S193F^b^; D225N
	I	D	K140I, S193F^b^; D225N
**2009**			
	I	D	
**2011**			
	I	D	E62K, N144K^b^, P162S, R261Q
	II	E	K140I; K158N; K173Q; N189K; S193F^b^; T212A
**2012**			
	II	E	K140I; K145N; K158N; K173Q; N189K; A198S; T212A; V223I;
	III	E	D53N^b^; K140I; K145S; K158N; K173Q; D188N; N189K; A198S; T212A; V223I
	IV	E	K140I; K145S; K158N; K173Q; N189K; A198S; T212A; V223I
	I	E	K140I; N144D; K145S; K158N; K173Q; N189K; T212A; V223I;
	V	E	D53N^b^; Y94H^b^; K140I; K145N; K158N; K173Q; N189K; S199A; T212A; I230V; E280A

^a^ Mutations present between 70 and 99 percent of sequences.

^b^ Codons under selective pressure.

As shown in Fig 1, clades A (in brown) and B (in purple) consisted exclusively of strains obtained during single epidemic seasons. Clade A included sequences obtained in winter 2004–2005 (December 2004-February 2005) and segregated into three subclades: two smaller subclades (I and II) with respectively two and four isolates, and a larger one consisting of 15 sequences (III). Clade B included sequences characterised by V112I, K173E and S199P amino acid variations that were obtained during February and March 2006 and grouped into a single highly significant clade. The few isolates obtained during January-March 2004 were at the root of the tree and represented two distinct clades of the global tree corresponding to A/Wellington/01/2004 and A/Wyoming/03/2003 genetic clades.

Clade C (in green) encompassed most of the strains obtained between January and March 2007, but also included two sequences isolated in January and February 2006, and consisted of one large subclade (III—14 isolates) and three small subclades (I, II and IV—three isolates each). The sequences belonging to subclade C-III were characterised by the amino acid change T128A, which generated the loss of an N-glycosylation site (NGS) in position 126.

Clade D (in red) included a majority of strains (n = 31) isolated between May and December 2009, and 12 sequences (n = 12) isolated between January and March 2007. These isolates were largely intermixed, and formed four small subgroups containing 3–6 isolates each, one of which included the five pure Italian isolates sampled from 2007 to 2009 found in the global analysis. Finally, there were two highly divergent strains obtained in January 2011 that shared a common node with a sequence obtained in May 2009: these two sequences were characterised by the amino acid changes E62K, N144K, P162S and R261Q.

Clade E (in blue) encompassed all of the sequences isolated between November 2011 and March 2012, and three sequences isolated during the preceding epidemic season (January 2011). The isolates obtained during the 2011–2012 epidemic season were further subdivided into five main groups (I-V), which corresponded to concurrently circulating viral mutants (Table 1), whereas the three isolates from the preceding epidemic seasons were at the outgroup of these subclades.

The estimated times of the most recent common ancestor (tMRCA) of the main clades are shown in Table 2.

**Table 2:** tMRCAs with credibility intervals (95%HPD).

CLADES	tMRCA^a^	LHPD^b^	UHPD^c^	DATE	Lower	Upper
**Tree root**	102	99	106	sep-03	dec-03	may-03
**A**	95	91	98	sep-04	aug-04	jan-04
**B**	84	79	91	mar-05	aug-05	aug-04
**C**	77	74	80	oct-05	jan-06	jul-05
**D**	68	64	71	jul-06	nov-06	apr-06
**E**	29	22	35	oct-09	may-10	apr-09

The corresponding months of the tree root and A(H3N2) Italian clades.

^a^tMRCA: Time of the most Recent Common Ancestor—month before present (March 2012).

^b^Lower 95% Highest Posterior Density.

^c^Upper 95% Highest Posterior Density.

The estimated tMRCA of the tree root was September 2003 (95% credibility interval: May 2003-December 2003). More detailed estimations of subclades TMRCAs are reported in S2 Table.

Clade A viruses circulating in winter 2004–2005 originated from an ancestor existing more than one year before (September 2004). Likewise, clade B, which included viral strains isolated during the 2005–2006 epidemic, probably originated from an ancestor existing since March 2005. Clade C, including strains that mainly circulated in the winter 2007, originated from an ancestor that appeared in October 2005, as is also suggested by the presence of two sequences isolated two years before (December 2005). The estimated tMRCA of clade D, which mainly contained strains circulating in 2009, dated back to July 2006 (more than two years before). The viral strains included in clade E, which mainly circulated in winter 2011–2012, had a common ancestor in October 2009, whereas the five subclades into which the isolates segregated had common ancestors dating back to between October and December 2010.

### Signature amino acid substitutions and HA selection pressure analysis

**Fig 2:**
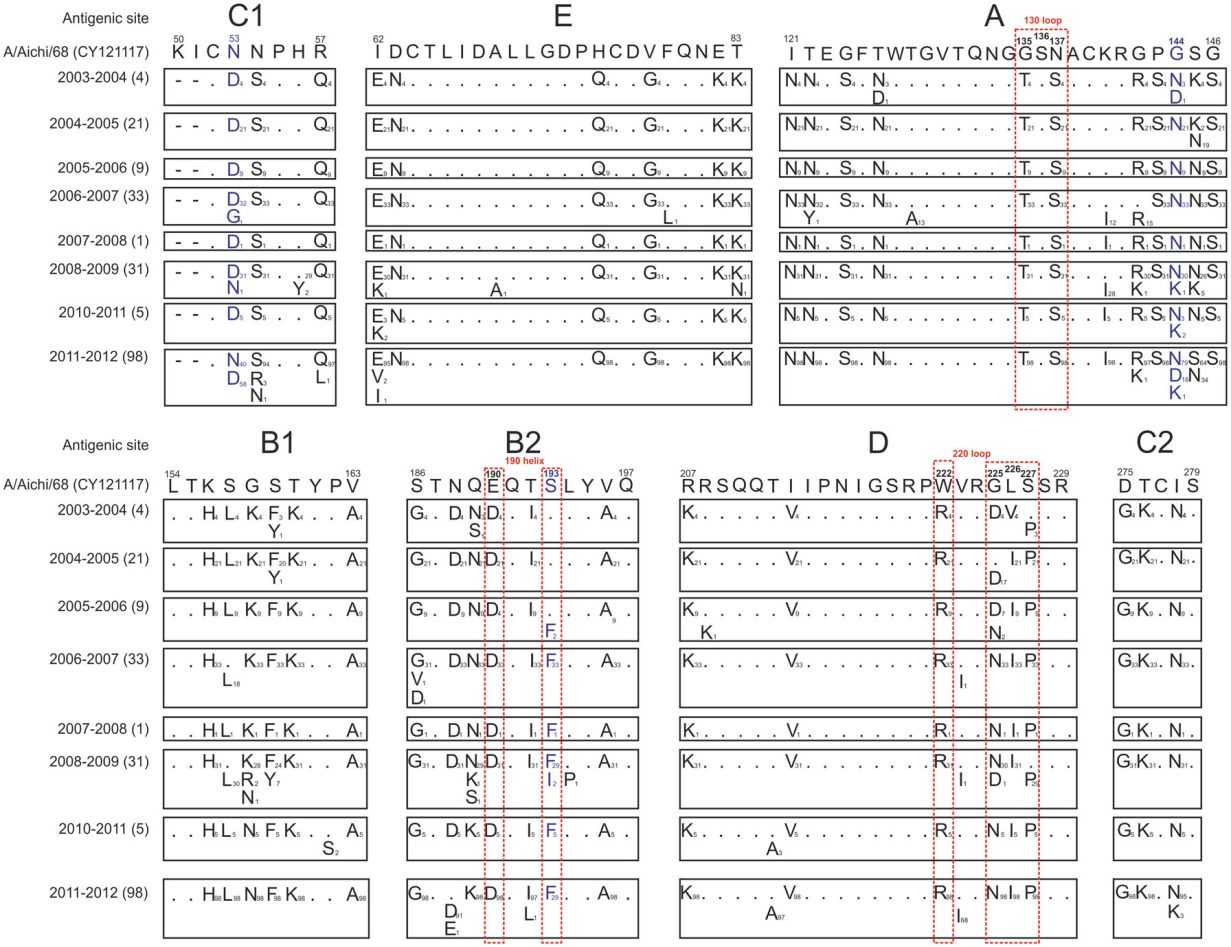
Amino acid changes in the antigenic sites domain in the A/Aichi/2/68(H3N2) influenza strain in eight consecutive seasons. The dots represent amino acids similar to those in the A/Aichi/2/68(H3N2) strain. The number of sequences analysed in each season is shown in brackets. The number of strains observed for each single amino acid substitution is shown in subscript.

The HA1 domain of HA contains all the H3 antigenic sites A-E. Fig 2 summarises the amino acid changes observed in the antigenic sites of the A/Aichi/2/1968(H3N2) virus. A total of 41/106 amino acid positions (38.7%) had at least one change, all of which were observed in the influenza strains circulating in the 2003–2004 season (Fig 1). However, a few key substitutions were observed during subsequent seasons: in 2005–2006, substitution S193F was accompanied by substitution D225N (Figs 1 and 2) in 2008–2009, a K158N substitution in antigenic site B1 was sporadically observed but became fixed in the following seasons; substitution T212A observed in HA sequences from season 2010–2011 was retained in sequences from the 2011–2012 season; and, in 2011–2012, a series of amino acid substitutions (D53N, N144D and N145S) were similar to those observed in the “older” A/Aichi/2/68 strain (in particular, substitution N144D generated the loss of an NGS site in position 144.

**Fig 3:**
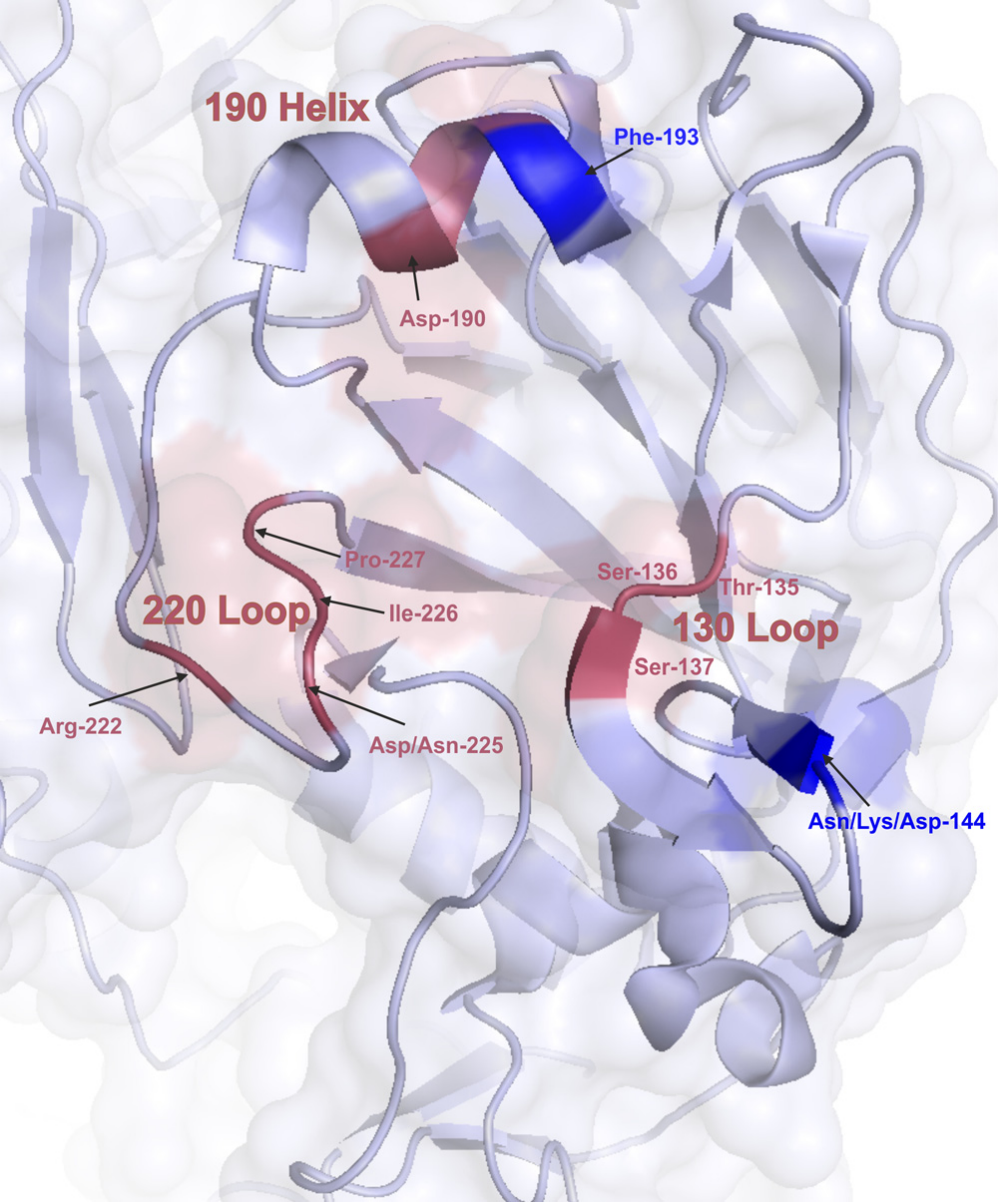
Structure of the receptor-binding site of the A/Aichi/2/68(H3N2) strain (PDB No. 3VUN). The amino acids involved in receptor-binding site are shown in red, and those under positive selection in blue. PyMol software was used to visualise the 3D structure.

A global analysis of selective pressure made using the SLAC model indicated an estimated overall dN/dS ratio of 0.33 and revealed no evidence of positive selection, whereas the FEL model identified three positively selected codons (53, 94 and 144) (Table 3). In addition, SLAC and FEL models have identified codons under negative selection, 11 and 29, respectively (data not showed). The alternative MEME model detected two sites under positive selection pressure (144 and 193). As shown in Figs 2 and 3, residue 144 is located in antigenic site A and residue 193 in the 190-helix of the receptor-binding site (RBS). The FUBAR method confirmed the positive selection of codon 144 and identified 33 sites as being under negative selection. The IFEL model was used to determine the selection pressure acting on the HA codons along the internal branches of tree. It identified six positively selected sites (codons 106, 112, 144, 189, 193 and 199) and nine codons under negative selective pressure. It is worth noting that codon 144 became an NGS in the globular domain of HA among A(H3N2) viruses in 1999 [17], and codons 189 and 193 belong to antigenic sites B2 near the 190-helix of the RBS.

**Table 3:** Selection pressure analysis.

Codon	SLACdN-dS	SLACp-value	FELdN-dS	FELp-value	MEMEω^+^	MEMEp-value	FUBARdN-dS	FUBARPost.Prob.
53	4.971	0.239	5.424	**0.094**	>100	0.122	0.41	0.899
94	4.422	0.285	5.537	**0.096**	>100	0.112	0.42	0.886
144	5.729	0.230	7.016	**0.061**	>100	**0.039**	0.679	**0.959**
193	3.984	0.316	5.073	0.108	>100	**0.010**	0.369	0.873

The analysis of 202 HA protein (231 codons) of A(H3N2) virus using SLAC, FEL, MEME and FUBAR methods (described in methods). The statistically significant values are reported in bold.

Finally, the PRIME method was used to estimate the biochemical properties preserved or modified by the evolutionary process. There were two residues (positions 170 and 173) with conserved properties, whereas codon 225 had changed properties.

### Population dynamics

**Fig 4:**
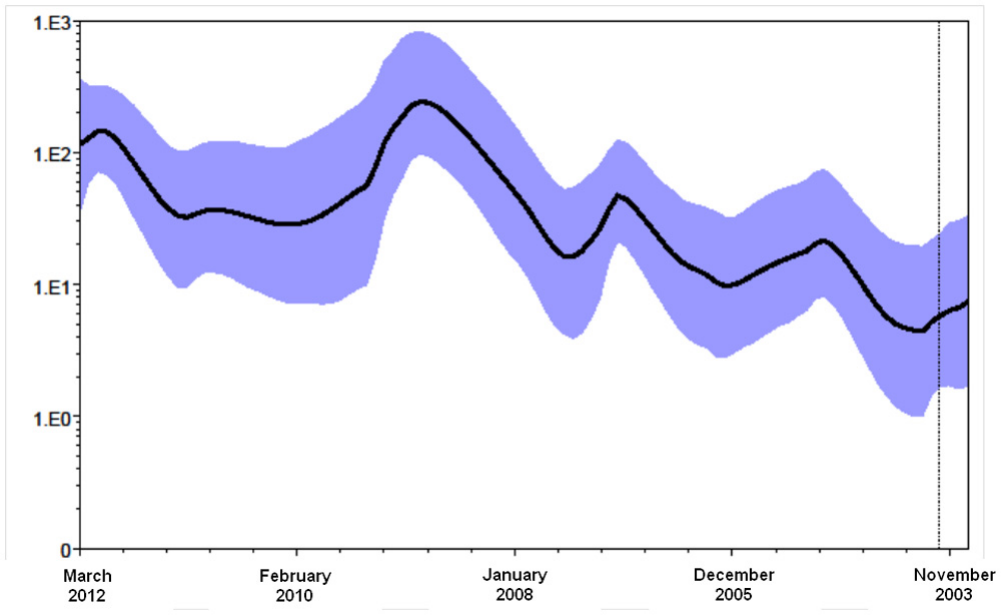
Bayesian skyride plot. Ordinate: the number of effective infections at time t (Ne(t)); abscissa: time before present in calendar month. Time = 0 (present) corresponds to the sampling month of the most recent isolates (March 2012). The thick solid line represents the median value, and the grey area the 95% HPD of the Ne(t) estimates. The vertical line indicates the 95% lower HPD tMRCA estimate of the tree root.

Analysis of the skyride curve indicating the size of the viral population in a calendar timescale (Fig 4) showed an alternation of periods of exponential growth (corresponding to the epidemic seasons 2004–2005, 2006–2007, 2008–2009, 2011–2012) with periods in which the number of infections decreased, corresponding to the inter-epidemic seasons. In particular, there was a long flat period between May 2009 and March 2011, corresponding to the circulation of the 2009 pandemic A(H1N1) influenza virus.

## Discussion

The activity and circulation of influenza viruses from 2004 to 2012 were investigated in the framework of the activities of the Italian Influenza Surveillance Network, and it was found that each influenza season was characterised by a specific virological profile: influenza virus type A detections predominated over type B during all of the considered epidemics except those that occurred in 2007–2008 and, within type A, the H3N2 subtype predominated over H1N1 in five of the eight seasons.

Significant proportions of influenza A(H3N2) viruses were detected during most of the analysed seasons (from 2003–2004 to 2006–2007, in 2008–2009 and 2011–2012), sporadically detected in the post-pandemic season (2010–2011), and not detected in the 2007–2008 season or during the 2009 pandemic, and the HA sequences of the isolated A(H3N2) viruses were analysed in order to reconstruct their dynamics in the light of the forces driving viral evolution. Our results show that at least five major A(H3N2) clades circulated in northern Italy between 2004 and 2012, each of which was subdivided into between one subclade (2008–2009) and five subclades (2011–2012) depending on the year. Subclades of exclusively Italian strains were rare as the majority of them included isolates from different locations, thus suggesting that the genetic heterogeneity of the Italian A(H3N2) virus was mainly due to multiple introductions of different strains during the same season.

Clades A, B and E harboured sequences that were only identified in a single season, whereas clades C and D also included sequences isolated in preceding and/or subsequent seasons, which explains why there were sometimes two or more clades co-circulating in the population. There were two clades during the 2005–2006 season: the first (clade B) was closely related to the A/California/07/2004 genetic group and entered Italy in summer 2005; the second (clade C), belonged to the A/Wisconsin/67/2005 genetic group. This last originated in the autumn of the same year, and became one of the two main clades circulating in the 2006–2007 season: the other was clade D, which was closely related to A/Brisbane/10/2007 and became dominant in 2008–2009. It is worth noting that the A(H3N2) viruses belonging to this latter clade were still circulating in 2010–2011, thus suggesting the possible persistence of a single strain for even several years.

There was a bottleneck before the 2011–2012 season because of the prevalent circulation of the A(H1N1)09 pandemic strain during the pandemic and post-pandemic (2009–2010 and 2010–2011) seasons [18]. A new influenza A(H3N2) virus belonging to the A/Victoria/208/2009 clade (clade E), which was characterised by a large number of genetic changes under positive selection, appeared in autumn 2009. The tMRCA estimates of the earliest branching events suggest that it probably entered Italy between September and December 2010, caused the winter 2011–2012 outbreak [19], and segregated into at least five subclades.

In general, the tMRCA estimates of the major clades suggest that a new viral strain originates several months or even years (6–31 months) before its effective circulation in the Italian population, thus supporting the importance of global migration in seeding the epidemics in Italy [20,21].

A total of 10 significant subclades of exclusively Italian isolates encompassed 3–15 sequences. Most of the Italian groups originated and became extinct in the same season, thus suggesting that they represent local epidemiological networks; however, a highly significant subclade (including five Italian isolates within Italian clade D) circulated from 2007 to 2009. This finding may of course be simply due to a casual absence of sequences of this clade from the international databanks but, more interestingly, it may indicate that influenza A viruses can sometimes “over-summer” also in the northern hemisphere and persist for a few years. The possible existence of local reservoirs capable of perpetuating viral circulation and supporting the year-round survival of a local strain even in temperate zones deserves more specific investigations [22–25].

The study of selection pressure showed that most of the amino acid substitutions distinguishing the viral clades and subclades were neutral. Only a minority were under positive selection, thus suggesting that the forces driving the local evolution of influenza A viruses are mainly stochastic. The estimated overall mean *dN/dS* ratio was in line with the values reported by others [26,27]. When estimating site-by-site rate variation, a SLAC counting method used to infer selection but this did not identify any sites under positive selection pressure. Our analysis showed that one codon (144) had signatures of episodic diversifying selection using both SLAC and MEME models, but codon 193 was only identified by the MEME model. Codon 193 is located in the 190-helix of the RBS but, as suggested by [28], substitution S193F is associated with a negligible impact on receptor binding activity. The IFEL method indicated the presence of six codons under positive selection pressure, thus suggesting a variation within the lineages: half of these codons were included in the antigenic sites. Finally, the PRIME analysis identified residue 225 as being under positive selection pressure. Additionally, the changes (D↔N/G) observed at the 225 site cause the changing of physicochemical properties of the residue. This position has also been reported to be responsible for altered receptor binding affinity [28].

Analysis of evolutionary population dynamics using a Bayesian skyride plot showed the typically fluctuating trend associated with the seasonal characteristic of influenza A outbreaks, with peaks corresponding to the major A(H3N2) epidemics. The greatest exponential growth was observed between summer 2007 and winter 2009, with a peak in November 2008 that was immediately followed by an evident bottleneck due to pandemic A(H1N1)09 virus in the following seasons. The epidemic resumed to grow in 2011, and reached a new peak in winter 2011–2012.

In conclusions, on the basis of our findings, it is clear that the main forces driving the local evolution of influenza A(H3N2) virus are stochastic [29], and mainly due to multiple founder effects. In any given season, different viral strains are imported from other geographical areas, and the epidemic is then amplified by local transmission networks, as in the case of A(H1N1)pdm09 [18]. This genetic drift leads to the loss of genetic heterogeneity within a population, but a large genetic distance between populations. This is particularly evident in season 2006–2007, when a viral variant (clade D) that probably entered Italy in July 2006 became the predominant strain, and was still being isolated in 2010–2011. During the same period, a purely Italian clade encompassed the seasons from 2007 to 2009.

Important selective sweeps sometimes cause a dramatic change in the predominant strain, such that observed during the 2011–2012 season. In this case, the absence of A(H3N2) viruses in previous seasons due to the prevalent circulation of the pandemic A(H1N1)09 strain caused the rise of a completely new strain that showed several positively selected sites. This complex evolutionary behaviour can be explained by interactions between the immune status of the population, migrations and selective sweeps [30].

The selection of a new strain characterised by multiple changes that allow it escape immune defences causes the rapid spread of infection within a susceptible population, but the consequent increase in the percentage of immunised subjects rapidly clears it. On the contrary, the rise of a strain with only minor neutral modifications in comparison with the previous season, and therefore circulating in a partially immune population, spreads more slowly and can persist longer at both global and local level. This seems to be confirmed by the observation that the most enduring strains are those showing the weakest selective pressure.

## Supporting Information

S1 Fig Likelihood mapping of the 202 A(H3N2) influenza virus HA gene sequences. Each dot represents the likelihoods of the three possible unrooted trees for each quartet randomly selected from the dataset: the dots near the corners or the sides respectively represent tree-like (fully resolved phylogenies where one tree is clearly better than the others) or network-like phylogenetic signals (three regions in which it is not possible to decide between the two topologies). The central area of the likelihood map represents a star-like signal (the region in which the star tree is the optimal tree). The numbers indicate the percentage of dots.
(TIF)

S2 Fig Root-to-tip regression of a Bayesian tree of the A(H3N2) influenza virus HA gene. (TIF)

S3 Fig Global phylogenetic tree of the 509 A(H3N2) influenza virus HA gene sequences: 202 from Italy, and 307 from the rest of the world. The Italian isolates are shown in red. The numbers on the internal nodes represent posterior probabilities.
(TIF)

S1 Table Names and identification numbers of the HA gene sequences used in constructing the global phylogenetic trees. (DOCX)

S2 Table tMRCAs with credibility intervals (95%HPD) and the corresponding months of the tree root and A(H3N2) Italian clades. (DOCX)
