# Supplementary figures and images for: Reconstruction of the Evolutionary Dynamics of A(H3N2) Influenza Viruses Circulating in Italy from 2004 to 2012
Source: PLoS One. 2015 Sep 2;10(9):e0137099. doi: 10.1371/journal.pone.0137099 (PMC4558001; doi:10.1371/journal.pone.0137099)

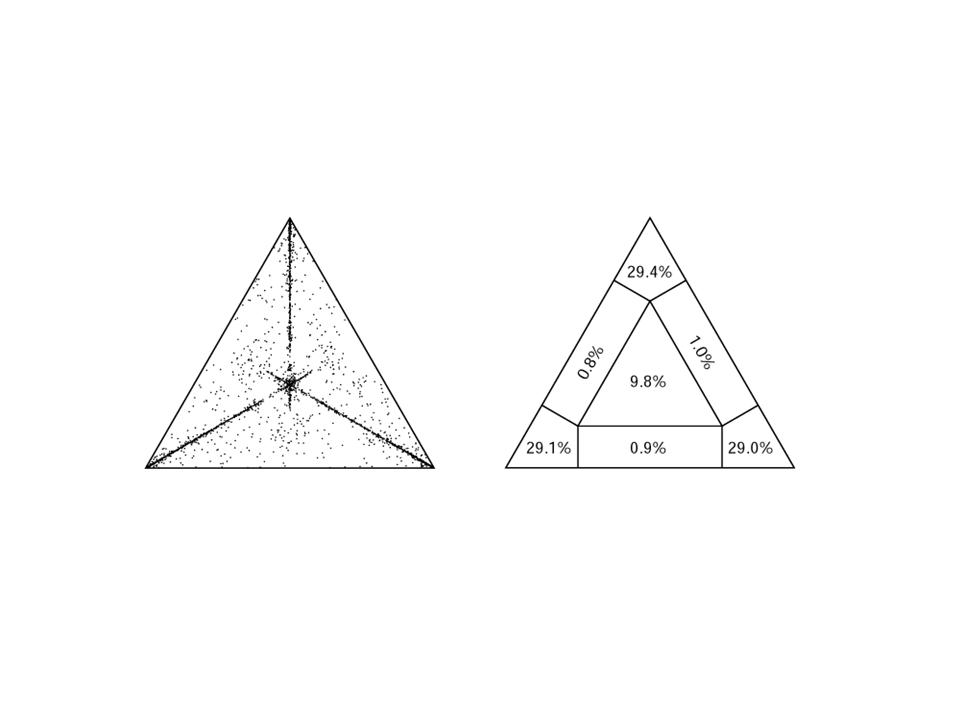

Supplement: S1 Fig — Each dot represents the likelihoods of the three possible unrooted trees for each quartet randomly selected from the dataset: the dots near the corners or the sides respectively represent tree-like (fully resolved phylogenies where one tree is clearly better than the others) or network-like phylogenetic signals (three regions in which it is not possible to decide between the two topologies). The central area of the likelihood map represents a star-like signal (the region in which the star tree is the optimal tree). The numbers indicate the percentage of dots. (TIF) [file pone.0137099.s001.tif]

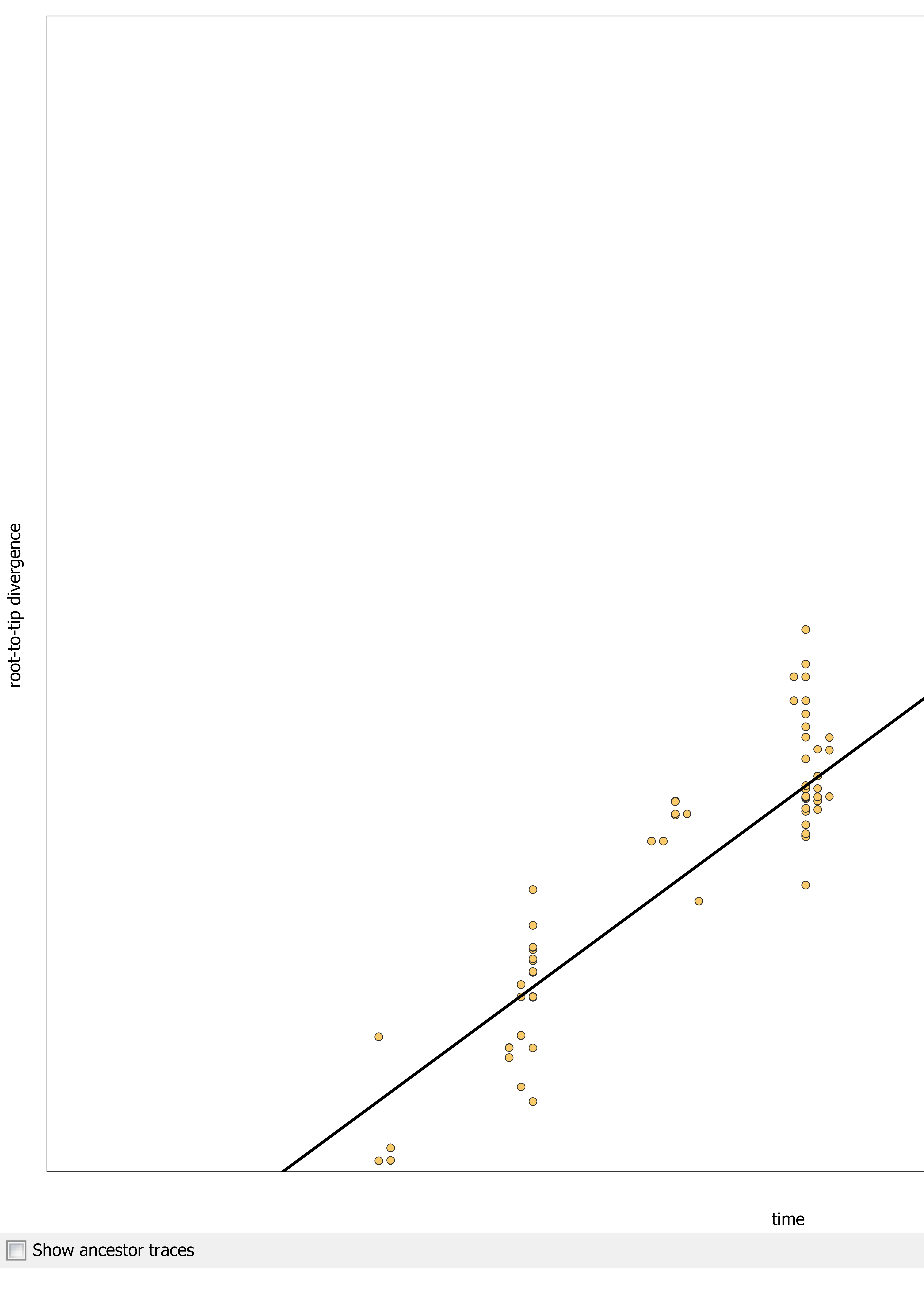

Supplement: S2 Fig — (TIF) [file pone.0137099.s002.tif]

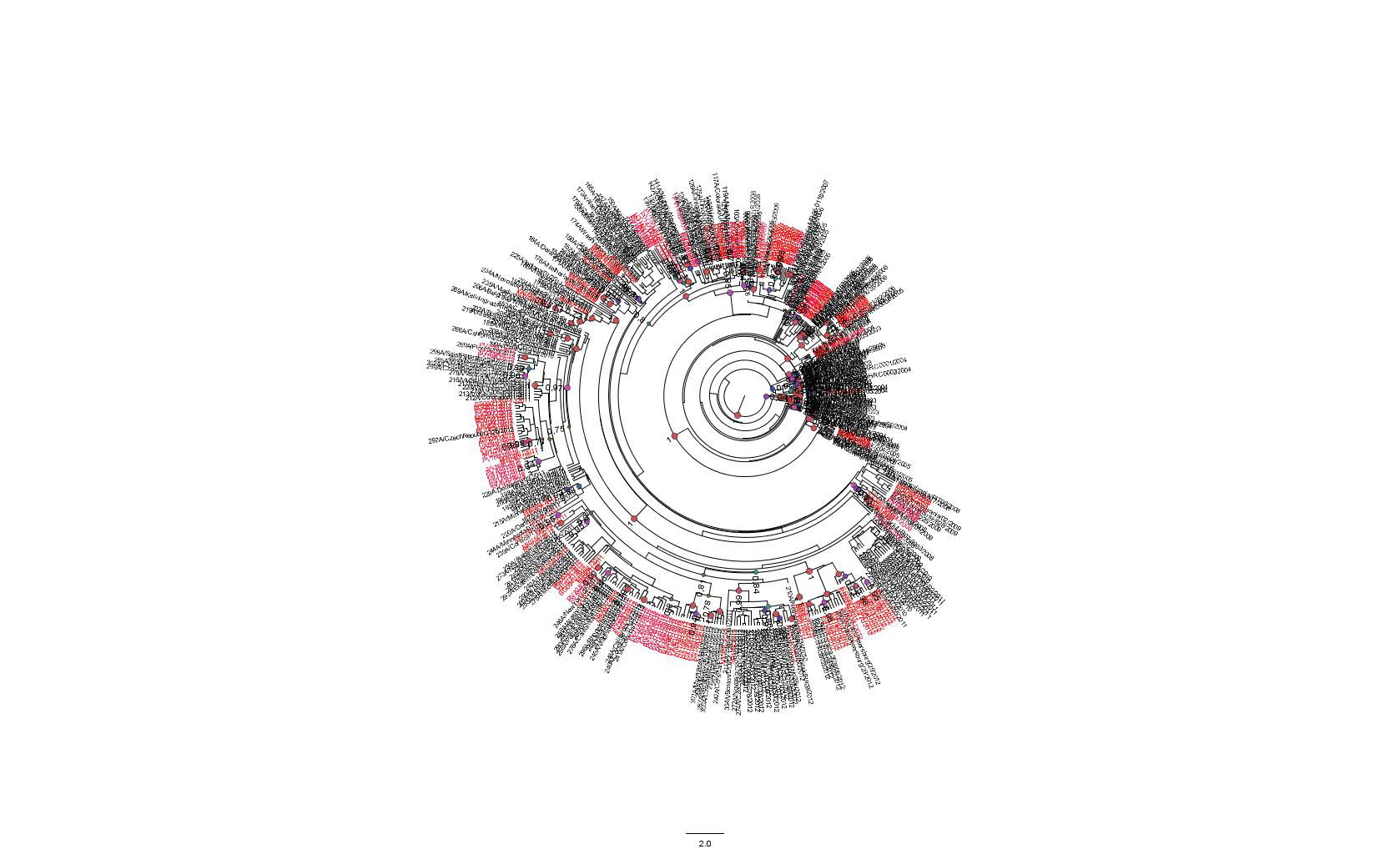

Supplement: S3 Fig — The Italian isolates are shown in red. The numbers on the internal nodes represent posterior probabilities. (TIF) [file pone.0137099.s003.tif]
