# Supplementary material for: Reconstruction of the Evolutionary Dynamics of A(H3N2) Influenza Viruses Circulating in Italy from 2004 to 2012
Source: PLoS One. 2015 Sep 2;10(9):e0137099. doi: 10.1371/journal.pone.0137099 (PMC4558001; doi:10.1371/journal.pone.0137099)
Supplement: S1 Table — (DOCX) [file pone.0137099.s004.docx]

|  | **NAME OF SEQUENCES** | **A.N.** |  |
| --- | --- | --- | --- |
| 1 | A/Denmark/59/2003 | AY531039 |  |
| 2 | A/Denmark/60/2003 | AY531044 |  |
| 3 | A/Denmark/61/2003 | AY531049 |  |
| 4 | A/Denmark/18-2/2003 | AY531059 |  |
| 5 | A/Moscow/343/2003 | DQ089637 |  |
| 6 | A/Moscow/328/2003 | DQ089636 |  |
| 7 | A/Moscow/346/2003 | DQ089639 |  |
| 8 | A/Denmark/113/2003 | EU103688 |  |
| 9 | A/Denmark/114/2003 | EU103743 |  |
| 10 | A/Denmark/108/2003 | EU103774 |  |
| 11 | A/Denmark/101/2003 | EU103816 |  |
| 12 | A/Wyoming/03/2003 | EU268227 |  |
| 13 | A/Finland/170/2003 | CY114349 |  |
| 14 | A/New York/485/2003 | CY003680 |  |
| 15 | A/New York/192/2003 | CY000753 |  |
| 16 | A/New York/194/2003 | CY000873 |  |
| 17 | A/Netherlands/20/2003 | CY112949 |  |
| 18 | A/New York/196/2003 | CY001536 |  |
| 19 | A/Netherlands/22/2003 | CY112957 |  |
| 20 | A/Netherlands/88/2003 | CY112965 |  |
| 21 | A/New York/197/2003 | CY001544 |  |
| 22 | A/Netherlands/109/2003 | CY112973 |  |
| 23 | A/New York/199/2003 | CY001253 |  |
| 24 | A/Netherlands/222/2003 | CY114357 |  |
| 25 | A/New York/214/2003 | CY006859 |  |
| 26 | A/Australia/NHRC0008/2003 | CY091277 |  |
| 27 | A/Queensland/31/2003 | CY018973 |  |
| 28 | A/New York/204/2003 | CY002520 |  |
| 29 | A/Queensland/33/2003 | CY017563 |  |
| 30 | A/Netherlands/312/2003 | CY114365 |  |
| 31 | A/England/431/2003 | CY087982 |  |
| 32 | A/Western Australia/44/2003 | CY015828 |  |
| 33 | A/Scotland/52/2003 | CY087990 |  |
| 34 | A/England/430/2003 | CY087976 |  |
| 35 | A/Scotland/61/2003 | CY088046 |  |
| 36 | A/New York/27/2003 | CY001112 |  |
| 37 | A/Scotland/72/2003 | CY088102 |  |
| 38 | A/England/425/2003 | CY107074 |  |
| 39 | A/New York/9/2003 | CY001285 |  |
| 40 | A/England/740/2003 | CY088062 |  |
| 41 | A/England/475/2003 | CY088110 |  |
| 42 | A/Scotland/71/2003 | CY088094 |  |
| 43 | A/England/460/2003 | CY088086 |  |
| 44 | A/England/438/2003 | CY088038 |  |
| 45 | A/South Carolina/NHRC0002/2003 | CY090949 |  |
| 46 | A/New York/270/2003 | CY001648 |  |
| 47 | A/England/516/2003 | CY088198 |  |
| 48 | A/New York/478/2003 | CY008900 |  |
| 49 | A/England/493/2003 | CY107154 |  |
| 50 | A/England/534/2003 | CY088374 |  |
| 51 | A/New York/35/2003 | CY000049 |  |
| 52 | A/England/567/2003 | CY088350 |  |
| 53 | A/New York/50/2003 | CY000089 |  |
| 54 | A/California/NHRC0003/2003 | CY091173 |  |
| 55 | A/Denmark/12/2004 | EU103658 |  |
| 56 | A/Denmark/113/2004 | EU103689 |  |
| 57 | A/Denmark/05/2004 | EU103710 |  |
| 58 | A/Denmark/102/2004 | EU103713 |  |
| 59 | A/Denmark/126/2004 | EU103814 |  |
| 60 | A/California/07/2004 | EU103820 |  |
| 61 | A/Missouri/NHRC0002/2004 | CY091117 |  |
| 62 | A/Georgia/NHRC0001/2004 | CY090973 |  |
| 63 | A/Western Australia/55/2004 | CY015908 |  |
| 64 | A/Western Australia/52/2004 | CY015884 |  |
| 65 | A/New York/470/2004 | CY006092 |  |
| 66 | A/Queensland/42/2004 | CY017957 |  |
| 67 | A/Queensland/50/2004 | CY020045 |  |
| 68 | A/New York/360/2004 | CY002424 |  |
| 69 | A/Netherlands/132/2004 | CY112989 |  |
| 70 | A/New York/371/2004 | CY002216 |  |
| 71 | A/New York/392/2004 | NC007371 |  |
| 72 | A/New York/236/2004 | CY003408 |  |
| 73 | A/Denmark/21/2005 | EU103646 |  |
| 74 | A/Denmark/152/2005 | EU103648 |  |
| 75 | A/Denmark/204/2005 | EU103671 |  |
| 76 | A/Denmark/201/2005 | EU103676 |  |
| 77 | A/Denmark/151/2005 | EU103696 |  |
| 78 | A/Denmark/32/2005 | EU103734 |  |
| 79 | A/Denmark/35/2005 | EU103753 |  |
| 80 | A/Denmark/178/2005 | EU103794 |  |
| 81 | A/South Australia/32/2005 | CY017821 |  |
| 82 | A/Denmark/68/2005 | EU103792 |  |
| 83 | A/New York/378/2005 | CY002016 |  |
| 84 | A/Western Australia/65/2005 | CY015988 |  |
| 85 | A/Missouri/NHRC0001/2005 | CY090981 |  |
| 86 | A/New York/361/2005 | CY002008 |  |
| 87 | A/California/NHRC0002/2005 | CY091429 |  |
| 88 | A/Texas/NHRC0001/2005 | CY091133 |  |
| 89 | A/Australia/NHRC0001/2005 | CY091421 |  |
| 90 | A/Australia/NHRC0012/2005 | CY091341 |  |
| 91 | A/Western Australia/66/2005 | CY015996 |  |
| 92 | A/Western Australia/68/2005 | CY016004 |  |
| 93 | A/Queensland/52/2005 | CY017989 |  |
| 94 | A/Wisconsin/67/2005 | CY164112 |  |
| 95 | A/Queensland/57/2005 | CY017619 |  |
| 96 | A/California/NHRC0004/2005 | CY091509 |  |
| 97 | A/Netherlands/548/2005 | CY112997 |  |
| 98 | A/Denmark/77/2006 | EU103655 |  |
| 99 | A/Denmark/60/2006 | EU103669 |  |
| 100 | A/Denmark/45/2006 | EU103677 |  |
| 101 | A/Denmark/81/2006 | EU103701 |  |
| 102 | A/Denmark/64/2006 | EU103738 |  |
| 103 | A/Denmark/100/2006 | EU103760 |  |
| 104 | A/Denmark/35/2006 | EU103800 |  |
| 105 | A/Denmark/82/2006 | EU103804 |  |
| 106 | A/Brisbane/09/2006 | CY121568 |  |
| 107 | A/Wyoming/02/2006 | EU199252 |  |
| 108 | A/Texas/NHRC0001/2006 | CY091037 |  |
| 109 | A/Illinois/NHRC0001/2006 | CY091045 |  |
| 110 | A/Netherlands/42/2006 | CY114389 |  |
| 111 | A/New York/938/2006 | CY020133 |  |
| 112 | A/South Carolina/NHRC0001/2006 | CY091061 |  |
| 113 | A/Vienna/28/2006 | JF340085 |  |
| 114 | A/Netherlands/363/2006 | CY114397 |  |
| 115 | A/Idaho/03/2007 | EU199275 |  |
| 116 | A/Brisbane/10/2007 | EU199366 |  |
| 117 | A/Colorado/UR06-022/2007 | CY027539 |  |
| 118 | A/Florida/01/2007 | EU100720 |  |
| 119 | A/New Mexico/01/2007 | EU199261 |  |
| 120 | A/Wisconsin/03/2007 | CY121528 |  |
| 121 | A/Indiana/01/2007 | EU199267 |  |
| 122 | A/California/UR06-0118/2007 | CY025739 |  |
| 123 | A/Missouri/02/2007 | EU199266 |  |
| 124 | A/Netherlands/69/2007 | CY114405 |  |
| 125 | A/Ohio/UR06-0410/2007 | CY027499 |  |
| 126 | A/Oregon/UR06-0450/2007 | CY026827 |  |
| 127 | A/Alaska/06/2007 | EU199374 |  |
| 128 | A/Michigan/07/2007 | EU516032 |  |
| 129 | A/Colorado/05/2008 | FJ686919 |  |
| 130 | A/Boston/92/2008 | CY044828 |  |
| 131 | A/Minnesota/05/2008 | EU885499 |  |
| 132 | A/Louisiana/05/2008 | EU851995 |  |
| 133 | A/Texas/01/2008 | EU716465 |  |
| 134 | A/Pennsylvania/PIT06/2008 | CY035046 |  |
| 135 | A/Maryland/03/2008 | EU716446 |  |
| 136 | A/Wisconsin/05/2008 | EU716469 |  |
| 137 | A/Mississippi/UR07-0004/2008 | CY036951 |  |
| 138 | A/New York/08/2008 | FJ549056 |  |
| 139 | A/Illinois/14/2008 | FJ549055 |  |
| 140 | A/New York/UR07-0093/2008 | CY037711 |  |
| 141 | A/Nizhniy Novgorod/668/2008 | JQ655462 |  |
| 142 | A/Ohio/UR07-0140/2008 | CY036999 |  |
| 143 | A/Alaska/04/2008 | FJ532066 |  |
| 144 | A/Netherlands/377/2008 | CY113013 |  |
| 145 | A/Wisconsin/18/2008 | FJ686928 |  |
| 146 | A/Montana/02/2008 | FJ686922 |  |
| 147 | A/Wisconsin/24/2008 | GQ369928 |  |
| 148 | A/Minnesota/36/2008 | GQ369860 |  |
| 149 | A/Uruguay/716/2007 | CY121632 |  |
| 150 | A/Kentucky/02/2009 | GQ385931 |  |
| 151 | A/Wisconsin/56/2009 | KC535406 |  |
| 152 | A/Finland/97/2009 | KF765561 |  |
| 153 | A/California/VRDL363/2009 | CY073757 |  |
| 154 | A/Moscow/402/2009 | HQ616671 |  |
| 155 | A/South Dakota/WRAIR1177P/2009 | CY069501 |  |
| 156 | A/Iowa/03/2009 | GQ385846 |  |
| 157 | A/Netherlands/69/2009 | CY113021 |  |
| 158 | A/Massachusetts/01/2009 | GQ385856 |  |
| 159 | A/Colorado/06/2009 | GQ385815 |  |
| 160 | A/Pennsylvania/02/2009 | GQ385891 |  |
| 161 | A/Victoria/502/2009 | FJ966245 |  |
| 162 | A/Texas/02/2009 | GQ385935 |  |
| 163 | A/Novosibirsk/31/2009 | CY053660 |  |
| 164 | A/Delware/WRAIR1240/2009 | CY069517 |  |
| 165 | A/Texas/WRAIR1558P/2009 | CY093343 |  |
| 166 | A/Florida/01/2009 | GQ895034 |  |
| 167 | A/Lipezk/225/2009 | JQ988048 |  |
| 168 | A/Washington/WRAIR1057P/2009 | CY069349 |  |
| 169 | A/Hawaii/10/2009 | GQ385854 |  |
| 170 | A/Moscow/24/2009 | JQ988024 |  |
| 171 | A/Arizona/11/2009 | GQ385909 |  |
| 172 | A/Novosibirsk/628/2009 | CY053666 |  |
| 173 | A/Alaska/WRAIR1145P/2009 | CY069461 |  |
| 174 | A/Washington/16/2009 | GQ385926 |  |
| 175 | A/California/VRDL262/2009 | CY068345 |  |
| 176 | A/Netherlands/761/2009 | CY113037 |  |
| 177 | A/Australia/2/2009 | CY061890 |  |
| 178 | A/Australia/46/2009 | CY080531 |  |
| 179 | A/Australia/55/2009 | CY080563 |  |
| 180 | A/New York/01/2010 | KC535393 |  |
| 181 | A/Iowa/119/2010 | CY121213 |  |
| 182 | A/Perth/10/2010 | CY121496 |  |
| 183 | A/Brisbane/11/2010 | CY121792 |  |
| 184 | A/Rhode Island/01/2010 | KC535478 |  |
| 185 | A/Connecticut/04/2010 | KC535413 |  |
| 186 | A/New York/05/2010 | KC535419 |  |
| 187 | A/Virginia/03/2010 | KC535433 |  |
| 188 | A/Netherlands/009/2010 | CY114501 |  |
| 189 | A/California/10/2010 | KC535428 |  |
| 190 | A/New York/20342/2010 | CY070967 |  |
| 191 | A/Stockholm/4/2010 | HQ315822 |  |
| 192 | A/California/16/2010 | KC535295 |  |
| 193 | A/Ohio/01/2010 | KC535480 |  |
| 194 | A/New York/06/2010 | KC882652 |  |
| 195 | A/Hawaii/15/2010 | KC882647 |  |
| 196 | A/Delaware/04/2010 | KC535491 |  |
| 197 | A/Michigan/05/2010 | KC535462 |  |
| 198 | A/Minnesota/10/2010 | KC882826 |  |
| 199 | A/California/18/2010 | KC882651 |  |
| 200 | A/Sydney/DD2-02/2010 | CY090877 |  |
| 201 | A/Pennsylvania/12/2010 | KC883191 |  |
| 202 | A/District of Columbia/WRAIR1753P/2010 | CY093383 |  |
| 203 | A/Sydney/DD2-01/2010 | CY090869 |  |
| 204 | A/Hawaii/18/2010 | KC882762 |  |
| 205 | A/California/19/2010 | KC883292 |  |
| 206 | A/Belgrade/WRAIR2379N/2010 | CY093391 |  |
| 207 | A/Vladivostok/11/2010 | JQ746702 |  |
| 208 | A/Wisconsin/14/2010 | KC883288 |  |
| 209 | A/Denmark/105/2010 | HQ880599 |  |
| 210 | A/Netherlands/034/2010 | CY114509 |  |
| 211 | A/Victoria/361/2011 | KC306165 |  |
| 212 | A/Colorado/01/2011 | KC883212 |  |
| 213 | A/Mississippi/01/2011 | KC883216 |  |
| 214 | A/Boston/DOA03/2011 | CY111142 |  |
| 215 | A/Minnesota/07/2011 | KC883120 |  |
| 216 | A/Massachusetts/03/2011 | KC883057 |  |
| 217 | A/Boston/DOA04/2011 | CY111150 |  |
| 218 | A/Kansas/01/2011 | KC882930 |  |
| 219 | A/Massachusetts/02/2011 | KC882752 |  |
| 220 | A/Denmark/22/2011 | JF327386 |  |
| 221 | A/New Jersey/02/2011 | KC882557 |  |
| 222 | A/Boston/DOA06/2011 | CY111166 |  |
| 223 | A/Boston/DOA09/2011 | CY111182 |  |
| 224 | A/North Dakota/02/2011 | KC882813 |  |
| 225 | A/Indiana/01/2011 | KC882979 |  |
| 226 | A/Minnesota/04/2011 | KC882953 |  |
| 227 | A/Nebraska/05/2011 | KC882488 |  |
| 228 | A/Minnesota/05/2011 | KC882955 |  |
| 229 | A/Delaware/03/2011 | KC882923 |  |
| 230 | A/Rhode Island/03/2011 | KC882591 |  |
| 231 | A/Nevada/04/2011 | KC883336 |  |
| 232 | A/Boston/DOA20/2011 | CY111246 |  |
| 233 | A/New York/05/2011 | KC883129 |  |
| 234 | A/Novosibirsk/76K/2011 | JN940427 |  |
| 235 | A/Vladivostok/10/2011 | JQ988045 |  |
| 236 | A/Astrakhan/RII65/2011 | CY114553 |  |
| 237 | A/Corsica/348502/2012 | KC814184 |  |
| 238 | A/Corsica/353906/2012 | KC814186 |  |
| 239 | A/Corsica/11/2012 | KC814190 |  |
| 240 | A/Corsica/F11104/2012 | KC814196 |  |
| 241 | A/Corsica/12/2012 | KC814200 |  |
| 242 | A/Corsica/F11604/2012 | KC814204 |  |
| 243 | A/Hawaii/01/2012 | KC892382 |  |
| 244 | A/Minnesota/02/2012 | KC893104 |  |
| 245 | A/Vladivostok/59/2012 | CY110774 |  |
| 246 | A/New York/02/2012 | KC893183 |  |
| 247 | A/Virginia/01/2012 | KC892806 |  |
| 248 | A/California/18/2012 | KC892364 |  |
| 249 | A/Maryland/02/2012 | KC893166 |  |
| 250 | A/Alabama/02/2012 | KC893090 |  |
| 251 | A/Washington/10/2012 | KC892352 |  |
| 252 | A/Novosibirsk/RII09/2012 | CY114558 |  |
| 253 | A/Saint-Petersburg/RII01/2012 | CY114563 |  |
| 254 | A/Tyumen/03/2012 | KC135512 |  |
| 255 | A/Corsica/02/2012 | KC814187 |  |
| 256 | A/Corsica/08/2012 | KC814189 |  |
| 257 | A/Moscow/02/2012 | JQ988033 |  |
| 258 | A/Petrozavodsk/RII01/2012 | CY114533 |  |
| 259 | A/Washington/11/2012 | KC892355 |  |
| 260 | A/Michigan/02/2012 | KC892477 |  |
| 261 | A/Saint-Petersburg/RII02/2012 | CY114543 |  |
| 262 | A/West Virginia/03/2012 | KC892326 |  |
| 263 | A/Moscow/03/2012 | JQ988036 |  |
| 264 | A/Moscow/RII05/2012 | KC488837 |  |
| 265 | A/Novosibirsk/26k/2012 | KC135502 |  |
| 266 | A/California/22/2012 | KC892829 |  |
| 267 | A/Illinois/02/2012 | KC892866 |  |
| 268 | A/Saint Petersburg/RII25/2012 | KC488815 |  |
| 269 | A/Florida/05/2012 | KC892482 |  |
| 270 | A/Corsica/01/2012 | KC814203 |  |
| 271 | A/Kansas/05/2012 | KC892450 |  |
| 272 | A/Novosibirsk/RII25/2012 | JX978737 |  |
| 273 | A/North Dakota/04/2012 | KC892517 |  |
| 274 | A/Czech Republic/121/2012 | JX913035 |  |
| 275 | A/Michigan/03/2012 | KC892838 |  |
| 276 | A/California/NHRC348923/2012 | KF182345 |  |
| 277 | A/Voroneg/RII03/2012 | KC488817 |  |
| 278 | A/Vermont/07/2012 | KC892485 |  |
| 279 | A/Novosibirsk/RII39/2012 | KC488830 |  |
| 280 | A/Saint Petersburg/RII23/2012 | JX978746 |  |
| 281 | A/Moscow/RII13/2012 | JX978782 |  |
| 282 | A/Czech Republic/76/2012 | JX913043 |  |
| 283 | A/Ekaterinburg/RII06/2012 | JX978770 |  |
| 284 | A/Alabama/07/2012 | KC893059 |  |
| 285 | A/Washington/2997/2012 | CY120882 |  |
| 286 | A/Kaliningrad/RII09/2012 | KC488843 |  |
| 287 | A/Saint Petersburg/RII51/2012 | KC488831 |  |
| 288 | A/Maine/05/2012 | KF789696 |  |
| 289 | A/Kaliningrad/RII10/2012 | KC488827 |  |
| 290 | A/Saint Petersburg/RII60/2012 | JX978776 |  |
| 291 | A/Saint Petersburg/RII50/2012 | JX978773 |  |
| 292 | A/Tennessee/07/2012 | KC892739 |  |
| 293 | A/Ekaterinburg/RII13/2012 | KC488834 |  |
| 294 | A/Texas/50/2012 | KC892952 |  |
| 295 | A/Irkutsk/RII04/2012 | KC488840 |  |
| 296 | A/Rhode Island/07/2012 | KC892667 |  |
| 297 | A/Czech Republic/126/2012 | JX913059 |  |
| 298 | A/Saint Petersburg/RII47/2012 | JX978767 |  |
| 299 | A/Czech Republic/114/2012 | JX913067 |  |
| 300 | A/Czech Republic/131/2012 | JX913072 |  |
| 301 | A/Murmansk/RII06/2012 | KC488828 |  |
| 302 | A/Czech Republic/138/2012 | JX913079 |  |
| 303 | A/Boston/DOA2-103/2012 | CY148348 |  |
| 304 | A/Boston/DOA2-128/2012 | CY148540 |  |
| 305 | A/New York/3212/2012 | CY141227 |  |
| 306 | A/Washington/3262/2012 | CY141277 |  |
| 307 | A/Boston/DOA2-169/2012 | CY148828 |  |
|  |  |  |  |
|  |  |  |  |
