# Supplementary material for: Reconstruction of the Evolutionary Dynamics of A(H3N2) Influenza Viruses Circulating in Italy from 2004 to 2012
Source: PLoS One. 2015 Sep 2;10(9):e0137099. doi: 10.1371/journal.pone.0137099 (PMC4558001; doi:10.1371/journal.pone.0137099)
Supplement: S2 Table — (DOCX) [file pone.0137099.s005.docx]

| **Season** | **CLUSTERS** | **ITALIAN CLADES** | **tMRCA^1^** | **LHPD^2^** | **UHPD^3^** | **DATE** | **Lower** | **Upper** |
| --- | --- | --- | --- | --- | --- | --- | --- | --- |
| 2003-2004 | I (root) | - | 102 | 99 | 106 | sep-03 | dec-03 | may-03 |
|  | II | - | 99 | 97 | 101 | dec-03 | feb-04 | oct-03 |
| 2005 | **root** | **A** | **95** | **91** | **98** | **sep-04** | **aug-04** | **jan-04** |
|  | II | A | 90 | 86 | 91 | sep-04 | jan-05 | aug-04 |
|  | I | A | 89 | 85 | 93 | oct-04 | feb-05 | jun-04 |
|  | III | A | 91 | 87 | 93 | aug-04 | dec-04 | jun-04 |
| 2006 | **root** | **B** | **84** | **79** | **91** | **mar-05** | **aug-05** | **aug-04** |
|  | I | B | 76 | 72 | 79 | nov-05 | mar-06 | aug-05 |
|  | **II** | **C** | **77** | **74** | **80** | **oct-05** | **jan-06** | **jul-05** |
| 2007 | III | C | 71 | 66 | 73 | apr-06 | sep-06 | feb-06 |
|  | II | C | 64 | 61 | 67 | nov-06 | feb-07 | aug-06 |
|  | IV | C | 69 | 65 | 72 | jun-06 | oct-06 | mar-06 |
|  | **I** | **D** | **68** | **64** | **71** | **jul-06** | **nov-06** | **apr-06** |
| 2008-2009 | I | D | 59 | 55 | 61 | apr-07 | aug-07 | feb-07 |
| 2010-2011 | I | D | 35 | 33 | 37 | apr-08 | jun-09 | feb-09 |
|  | II | E | 26 | 19 | 32 | jan-10 | aug-10 | jul-09 |
| 2011-2012 | **root** | **E** | **29** | **22** | **35** | **oct-09** | **may-10** | **apr-09** |
|  | II | E | 7 | 2 | 11 | aug-11 | jan-12 | apr-11 |
|  | III | E | 8 | 3 | 12 | jul-11 | dec-11 | mar-11 |
|  | IV | E | 15 | 9 | 20 | dec-10 | jun-11 | jul-10 |
|  | I | E | 17 | 10 | 23 | oct-10 | may-11 | apr-10 |
|  | V | E | 15 | 8 | 21 | dec-10 | jul-11 | jun-10 |

| ^1^ tMRCA: Time of the most Recent Common Ancestor | | |
| --- | --- | --- |
| ^2^ Lower 95% Highest Posterior Density |  |  |
| ^3^ Upper 95% Highest Posterior Density |  |  |
